# 

**Published:** 1879-02-20

**Authors:** 


VOL. IX. FEBRUARY 20, 1879. NO. 2.

### THE SOUTHERN·MEDICAL RECORD:

S.G.O. INDEXED

A MONTHLY JOURNAL OF PRACTICAL MEDICINE.

Terms: $2.00 per Annum, in Advance; 4 Copies $6.00; Single Copies 20 Cents.

*"QUICQUID PRÆCIPIES ESTO BREVIS."*

Address R. C. WORD, M.D., Managing Editor, Atlanta, Ga.

